# Association analysis for udder health based on SNP-panel and sequence data in Danish Holsteins

**DOI:** 10.1186/s12711-015-0129-1

**Published:** 2015-06-19

**Authors:** Xiaoping Wu, Mogens S Lund, Goutam Sahana, Bernt Guldbrandtsen, Dongxiao Sun, Qin Zhang, Guosheng Su

**Affiliations:** Center for Quantitative Genetics and Genomics, Department of Molecular Biology and Genetics, Aarhus University, DK-8830 Tjele, Denmark; Key Laboratory of Animal Genetics and Breeding of Ministry of Agriculture, National Engineering Laboratory for Animal Breeding, College of Animal Science and Technology, China Agricultural University, Beijing, 100193 China

## Abstract

**Background:**

The sensitivity of genome-wide association studies for the detection of quantitative trait loci (QTL) depends on the density of markers examined and the statistical models used. This study compares the performance of three marker densities to refine six previously detected QTL regions for mastitis traits: 54 k markers of a medium-density SNP (single nucleotide polymorphism) chip (MD), imputed 777 k markers of a high-density SNP chip (HD), and imputed whole-genome sequencing data (SEQ). Each dataset contained data for 4496 Danish Holstein cattle. Comparisons were performed using a linear mixed model (LM) and a Bayesian variable selection model (BVS).

**Results:**

After quality control, 587, 7825, and 78 856 SNPs in the six targeted regions remained for MD, HD, and SEQ data, respectively. In general, the association patterns between SNPs and traits were similar for the three marker densities when tested using the same statistical model. With the LM model, 120 (MD), 967 (HD), and 7209 (SEQ) SNPs were significantly associated with mastitis, whereas with the BVS model, 43 (MD), 131 (HD), and 1052 (SEQ) significant SNPs (Bayes factor > 3.2) were observed. A total of 26 (MD), 75 (HD), and 465 (SEQ) significant SNPs were identified by both models. In addition, one, 16, and 33 QTL peaks for MD, HD, and SEQ data were detected according to the QTL intensity profile of SNP bins by post-analysis of the BVS model.

**Conclusions:**

The power to detect significant associations increased with increasing marker density. The BVS model resulted in clearer boundaries between linked QTL than the LM model. Using SEQ data, the six targeted regions were refined to 33 candidate QTL regions for udder health. The comparison between these candidate QTL regions and known genes suggested that *NPFFR2*, *SLC4A4*, *DCK*, *LIFR*, and *EDN3* may be considered as candidate genes for mastitis susceptibility.

**Electronic supplementary material:**

The online version of this article (doi:10.1186/s12711-015-0129-1) contains supplementary material, which is available to authorized users.

## Background

Mastitis, i.e. inflammation of the mammary gland, is a common and costly disease [1, 2] that is particularly problematic in the dairy industry. It adversely affects both animal and human health, since milk from affected cattle can enter the food supply and pose a health risk [3]. Mastitis also leads to economic losses owing to reduction in milk yield and quality, increased costs associated with treatment of affected cattle, discarded milk, and culling of severely affected cattle [4]. Therefore, prevention of mastitis is an extremely important breeding goal for dairy cattle. However, the heritability of clinical mastitis is low [5–9], and its genetic correlation with production traits is unfavorable [6, 10, 11]. Identification of specific genes associated with clinical mastitis would be extremely useful in breeding programs to reduce mastitis incidence. To that end, quantitative trait loci (QTL) mapping is a useful tool to identify genomic regions that are associated with clinical mastitis. Genome-wide association studies (GWAS) have been successfully used to identify QTL regions for a variety of traits in livestock [12]. Several studies have also attempted to detect QTL that affect clinical mastitis, with varying success [13–16].

Many factors influence the efficiency of GWAS to detect QTL. One of the most important factors is marker density. Currently, a medium-density (MD) SNP chip with ~54 000 markers is widely used for GWAS in dairy cattle [17–19]. In recent years, two high-density (HD) SNP chips with 777 962 SNPs from Illumina Inc. [20] and 648 874 SNPs from Affymetrix Inc. [21], and whole-genome sequencing (SEQ) data [22] have become available. Higher marker densities mean that markers are in stronger linkage disequilibrium (LD) with QTL that affect the trait of interest. In addition, genome sequencing data includes causative genetic variants. Thus, it is expected that using HD or SEQ markers will lead to the detection of more and more accurately localized QTL.

In addition to marker density, the statistical model selected for analysis has an effect on detection sensitivity. Several models have been proposed for GWAS, such as single-marker tests (SMT) [23], mixed model analysis (MMA) and linear models (LM) [24, 23], haplotype models (HM) [25], genealogy-based mixed-model (GENMIX) [25], and Bayesian variable selection models (BVS) [26]. Some studies have carried out model comparisons using simulated data. For example, Sahana et al. [23] compared the SMT, HM, LM, and BVS models, and concluded that the BVS model performed best. Dashab et al. [25] compared LM, random HM, GENMIX, and BVS models, and showed that LM and BVS were better than the other methods in terms of power and type-I error rate. However, there are very few reports on model comparisons based on real data from livestock [27, 28].

In general, LM models are performed in single-marker test analyses, for which each SNP is fitted separately in the model. In contrast, BVS models estimate the effects of all SNPs simultaneously. Usually, BVS models are implemented via Markov chain Monte Carlo (MCMC) algorithms [29–31]. For both models, false positive associations due to population structure, such as family relationships can be controlled [23, 24] by including systematic factors and taking polygenic effects into account. For LM models, significant associations are commonly established using a t-test with Bonferroni correction, assessment of false discovery rate, or permutation testing [32]. For BVS models, the number of QTL can be determined by post-MCMC analysis using Bayes factors [33] or QTL intensity profiles [29]. Previous studies have claimed that BVS models yield higher power than LM and are a solution to the problem of establishing significance of multiple-testing in simulation studies [25, 23]. Sahana et al. [23] compared the marginal posterior probability for single markers (BAYSM) and the joint posterior probabilities for intervals of 11 markers (BAYINT) to infer the presence of QTL using a BVS model based on simulated data. They reported that BAYINT resulted in higher power to detect QTL, while BAYSM was more precise in estimating QTL position.

Complex traits are likely influenced by multiple QTL that have small individual effects but a large collective effect on the phenotype. Conversely, the effects of a given QTL can be distributed over several markers that are in LD with the QTL by using a Bayesian model which estimates effects of all SNPs simultaneously. The QTL intensity profile, which summarizes the marginal posterior probabilities of markers in a small region, was proposed to detect QTL regions [34]. In this situation, a small QTL can be detected via a marked peak, and multiple QTL can be separated [34]. QTL intensity profiles based on post-MCMC analysis provide a suitable approach to identify QTL regions using real data.

The first objective of this study was to refine six previously detected QTL regions for udder health in Danish Holstein cattle using three marker densities, i.e. MD, HD and SEQ datasets. The second objective was to compare the performance of the LM and BVS models to detect and separate QTL that are closely located.

## Methods

### Phenotype and genotype data

A total of 4496 Danish Holstein bulls with de-regressed estimated breeding values (DRP) for the udder health index were used. The index for udder health is a measure of the genetic value of the cow’s resistance to mastitis. The index was calculated based on estimated breeding values (EBV) of clinical mastitis (CM) from the 1^st^ to 3^rd^ lactations, i.e.:

$$ \mathrm{Index}\ \mathrm{of}\ \mathrm{udder}\ \mathrm{health} = 0.25*{\mathrm{CM}}_{11} + 0.25*{\mathrm{CM}}_{12} + 0.30*{\mathrm{CM}}_2 + 0.20*\mathrm{C}\mathrm{M}3, $$

where CM_11_, CM_12_, CM_2_, CM_3_ are EBV for clinical mastitis in lactation 1 from days in milk (DIM) -15 (15 days before calving) to 50, lactation 1 from 51 to 305 DIM, lactation 2 from −15 to 150 DIM, and lactation 3 from −15 to 150 DIM, respectively. Cows with and without clinical mastitis were recorded as 1 and 0, respectively. Breeding values of clinical mastitis for each lactation were estimated using a multi-trait random regression test-day animal model. Somatic cell counts for the 1^st^, 2^nd^ and 3^rd^ lactation, fore udder attachment, and udder depth from the 1^st^ lactation were included as correlated traits to improve the accuracy of EBV for clinical mastitis [35], but EBV of these correlated traits were not included in the index of udder health. Udder health DRP [36, 37] were derived from the index of udder health evaluated in November 2010.

An association study for udder health was carried out using three marker datasets: (1) Illumina BovineSNP50 BeadChip (MD), (2) Illumina BovineHD BeadChip (HD), and (3) genome sequence data (SEQ). Six chromosome regions with significant effects on clinical mastitis that were reported in our previous study [38], were used for further association analysis. These six regions are located between 84 and 95 Mb on BTA5 (BTA for *Bos taurus* chromosome), 88 and 96 Mb on BTA6, 57 and 63 Mb on BTA13, 48 and 55 Mb on BTA16, 55 and 58 Mb on BTA19, and 32 and 40 Mb on BTA20. SNP array genotyping, sequence analysis, and imputation were performed as described by Sahana et al. [38]. Briefly, 5214 bulls were genotyped with the 50 k SNP chip. SNP chip data were edited using a number of criteria. First, individuals with a call rate higher than 85 % and SNPs with a call rate higher than 95 % were kept. SNPs with a minor allele frequency below 5 % and those that deviated from the Hardy-Weinberg equilibrium (P < 0.00001), or with average GenCall scores (Illumina Inc.) below 0.65 were excluded. After this quality control filtering, 43 415 SNPs remained. The MD data of 5214 Holstein bulls were imputed to HD data with BEAGLE [39], using 557 HD genotyped bulls (including 138 Nordic Holstein bulls, which represent a substantial contribution of the population) in the EuroGenomics project [40] as reference data. After quality control (using criteria similar to those described above for the 50 k chip), 648 219 HD SNPs remained. Finally, six regions with 790 SNPs from the MD chip and 8260 SNPs from the HD chip were targeted for analysis. Linkage disequilibrium (average r^2^ between adjacent marker pairs) estimates were equal to 0.21 for the MD and 0.56 for the HD data in the six targeted regions.

Sequence data analysis was performed at the Center for Quantitative Genetics and Genomics at Aarhus University. Details on sequencing, sequence analysis, and quality control are in [41, 42, 38]. Using the 89 sequenced bulls (including 32 Nordic Holstein bulls, which represent a substantial contribution of the present population) as reference data, 5214 Holstein bulls with imputed HD data for six targeted regions were imputed to SEQ data by BEAGLE [39]. A total of 196 882 SNPs were located within the six regions for the SEQ data. In the end, using all available phenotypic and genotypic data, 4496 Holstein bulls with MD, imputed HD, and imputed SEQ data were used in the analyses. These animals were the progeny of 373 bulls with 3169 dams.

The SNPs in each of the six regions were checked for quality as follows. First, SNPs that exclusively appeared in lower density data but disappeared in higher density data were discarded, removing 201 SNPs that were in MD but not in the HD and SEQ data as well as 308 SNPs that were in the HD but not in the SEQ data. Second, SNPs with a minor allele frequency less than 0.01 were discarded, removing one SNP from the HD dataset and 41 304 SNPs from the SEQ dataset. Finally, if two markers were in complete LD, then either the SNP that was absent from the lower marker density dataset was deleted or the more downstream SNP was deleted if both markers were absent from the lower density data. This removed two SNPs in the MD, 126 SNPs in the HD, and 76 722 SNPs in the SEQ data. After these edits, 587, 7825, and 78 856 SNPs in the six regions remained in the MD, HD, and SEQ data, respectively. The average distances between adjacent SNPs were approximately 70.0, 5.5, and 0.5 kb for the MD, HD, and SEQ data, respectively.

### Statistical analyses

A linear mixed model and a Bayesian variable selection model were used to detect associations of SNPs with phenotypic data, as described below.

#### Linear mixed model (LM)

The LM model [24] used in this study is a single-SNP regression model. The model includes a fixed regression of phenotypes using SNP genotypes as a measure of the SNP effects. In addition, a random polygenic effect that accounts for shared genetic effects of related individuals was included. The statistical model is described by the formula:

1 $$ \mathbf{y}=\mathbf{1}\mu + \mathbf{x}\mathrm{g} + \mathbf{Z}\mathbf{u}+\mathbf{e}, $$

where **y** is the vector of phenotypes (DRP), **1** is a vector of ones, μ is the overall mean, g is the additive genetic effect of the analyzed SNP, **x** is a vector of SNP genotypes (coded as 0, 1 or 2), and **u** is a vector of random polygenic effects, which are normally distributed **u** ~ N(0, **A***σ*_*u*_^2^), where **A** is the pedigree-based additive relationship matrix, *σ*_*u*_^2^ is the polygenic variance, **Z** is an incidence matrix relating phenotypes to the corresponding random polygenic effects, and **e** is a vector of residual effects, which are normally distributed **e** ~ N(0, Dσ_*e*_^2^), where **D** is a diagonal matrix with elements d_*ii*_ = (1 − *r*_*DRP*_^2^)/r_*DRP*_^2^ to account for heterogeneous residual variances due to different reliabilities of DRP (*r*_*DRP*_^2^), and *σ*_*e*_^2^ is the residual variance.

Significance testing of SNP effects was performed using a two-sided t-test. Our aim was to refine QTL regions from the selected large regions using the same population that was used to identify these regions. Although the segregation of QTL in the targeted regions was known, regions were large (up to 11 Mb). Therefore, we assumed that, except for a few, most of the SNPs were not associated with the trait. Accordingly, our null hypothesis was g = 0. A Bonferroni correction was applied to control for false positive associations. We declared a SNP significant if the *P* value was less than 0.05/*N*, where *N* is the number of SNPs. Therefore, the significant threshold value for − log_10_(*P*) was 4.07, 5.19, and 6.20 for the MD, HD, and SEQ data, respectively. Analyses were performed using the DMU package [43].

#### Bayesian variable selection model (BVS)

The BVS model [26, 44] used in this study describes SNP effects as a mixture distribution that estimates the effects of all SNPs simultaneously. The model is given by the following formula:

2 $$ \mathbf{y} = \mathbf{1}\upmu + {\displaystyle {\mathbf{\sum}}_{k=1}^m}{\mathbf{x}}_k{g}_k + \mathbf{Z}\mathbf{u} + \mathbf{e} $$

where **y**, **1**, μ, **u**, **Z**, and **e** are defined as in the LM model. The term ${\displaystyle {\mathbf{\sum}}_{k=1}^m}{\mathbf{x}}_k{g}_k$ fits additive SNP association effects, **x**_*k*_ is a vector of SNP genotypes (0, 1 or 2), and *g*_*k*_ is the effect of SNP k, which was assumed to follow a mixture distribution that assumes that most SNPs have a small phenotypic effect and some SNPs have a large effect:

3 $$ {\mathrm{g}}_k\sim \left\{\begin{array}{c}\hfill \mathrm{N}\left(0,\ {\sigma}_{g_0}^2\right)\ \mathrm{with}\ \mathrm{probability}\ {\pi}_0\hfill \\ {}\hfill \mathrm{N}\left(0,\ {\sigma}_{g_1}^2\right)\ \mathrm{with}\ \mathrm{probability}\ {\pi}_1=1-{\pi}_0\hfill \end{array}\right., $$

where *π*_0_ is the probability of a SNP having a small effect (i.e., drawn from a distribution with a small variance) and *π*_1_ is the probability of a SNP having a large effect (i.e., drawn from a distribution with a large variance). We assumed that the proportions in the two mixture distributions had a Bernoulli distribution and the prior distribution of *π*_0_ and *π*_1_ is a Beta (100, 1) distribution. We set *π*_0_ = 0.98, 0.998, and 0.9998 as priors for the MD, HD, and SEQ data, respectively. We also assumed that the priors μ, ${\sigma}_{g_0}^2$, and ${\sigma}_{g_1}^2$ followed a uniform distribution. By assuming a small variance instead of zero for the first distribution ($\mathrm{N}\left(0,\ {\sigma}_{g_0}^2\right)$), the MCMC implementation is straightforward with recognizable conditional distributions for all model parameters, as described elsewhere [26, 44]. Analysis of the BVS model was performed using the Bayz software [45]. The Gibbs sampler was run as a single chain with 52 000 samples. The first 20 000 iterations were discarded as burn-in. After this, every 20^th^ sample of the remaining 32 000 was saved for posterior analysis.

Within the Bayesian framework, the association of each SNP with the trait can be evaluated by a Bayes factor (BF). In this study, the BF for SNP *i* was calculated as the ratio between the estimated posterior probability and the average of posterior probabilities of SNPs with large effects (large variance, ${\mathrm{g}}_k\sim \mathrm{N}\left(0,\ {\sigma}_{g_1}^2\right)$) [46]:

4 $$ \mathrm{B}{\mathrm{F}}_i = \frac{{\hat{p}}_i/\left(1-{\hat{p}}_i\right)}{{\hat{\pi}}_1/\left(1-{\hat{\pi}}_1\right)}, $$

where ${\hat{p}}_i$ is the posterior probability of the effect for SNP *i* being drawn from the distribution with large effects, and ${\hat{\pi}}_1$ is the average of posteriors probabilities of SNPs with large effects. According to commonly used criteria [33], a BF between 3.2 and 10 was considered moderate and a BF greater than 10 as strong evidence for the segregation of a QTL affecting a trait.

#### QTL regions

A QTL region was detected using the posterior QTL intensity function [29]. In a Bayesian analysis, each chromosome is divided into many small intervals of equal length (bins), i.e. for instance, 1 or 2 cM in simulation studies [29, 47]. In our study, we defined the QTL intensity for each SNP on interval Δ_*i*_ as follows [29]:

5 $$ \mathrm{I}(i) = \frac{1}{N}\left[{\displaystyle {\sum}_{k=1}^N}\left({\displaystyle {\sum}_{q=1}^{N_{snp}^{(k)}}}{1}_{\left\{sn{p}_q^{(k)}\in \mathrm{T}\mathrm{S}\mathrm{D}\right\}}/{\Delta}_i\right)\right], $$

where *i* is the *i*^th^ bin, *N* is the number of MCMC samples kept for analysis, and ${1}_{\left\{sn{p}_q^{(k)}\in \mathrm{T}\mathrm{S}\mathrm{D}\right\}}$ is an indicator function with value 1 if the SNP in the *k*^th^ sample falls in the distribution with large variance (TSD), and Δ_*i*_ is the size of bin (constant number of markers in the current study) beginning from the *i*^th^ position. To avoid having too many SNPs diluting the QTL effect and to balance different marker densities, the bin size was set to 14, 18 and 18 SNPs, which correspond to 1, 0.1, and 0.01 Mb for the MD, HD, and SEQ data, respectively. The QTL intensity for each bin was calculated as the average posterior probability of SNPs having a large effect within the bin.

The QTL intensity profile within a region is expected to show a peak if the region contains a QTL. Xu et al. [34] declared that the intensity profile is not able to distinguish two linked QTL regions and that the QTL effect profile is not able to accurately locate the causative mutation within an QTL region, while an intensity profile [48] that is weighted by estimated SNP effects can distinguish intervals with QTL and also show sharp peaks within intervals. Therefore, we also used the weighted QTL intensity profile that has been used in several previous studies [49, 48, 34]. The weighted QTL intensity is defined as:

6 $$ {\mathrm{I}}_{\mathrm{w}}(i) = \mathrm{I}(i)*\mathrm{w}(i) $$

where I(*i*) is the QTL intensity of *i*^th^ bin, as given in Equation (5), and w(*i*) is the average SNP effect for the *i*^th^ bin.

To detect QTL regions based on the weighted QTL intensity profile, bins that were three standard deviations above or below the mean of total bins were deleted as outliers (assuming that the bins are in a QTL region) and the mean and standard deviation of the remaining bins were re-calculated for the purpose of the t test. Then, a multiple t-test $\Big(\mathrm{t} = \left(\mathrm{I}(i)\overline{-\mathrm{I}(i)}\right)/s$, where *s* is the standard deviation of the weighted QTL intensity of the bins) with a Bonferroni correction, was used to identify significant peaks among all the bins, including those outlier bins. Each significant peak represented a QTL region. A QTL region was specified as a region where the sum of the QTL intensities for the bins around the significant peak exceeded a predefined threshold (0.95). Finally, the average r^2^ (LD) between significant SNPs (BF ≥ 3.2) within a QTL region was calculated for each significant peak and between adjacent significant peaks. Adjacent peaks with an average r^2^ larger than 0.5 were merged into one region.

The position of each QTL region was defined according to the *Bos taurus* genome assembly UMD3.1 [50]. Genes that were located within or overlapped with the QTL regions were determined using information from the National Center for Biotechnology Information [51].

In the following, LM models with MD, HD, and SEQ data were designated LM_MD_, LM_HD_, and LM_SEQ_; BVS models with MD, HD, and SEQ data were designated BVS_MD_, BVS_HD_, and BVS_SEQ_; and BVS models using the QTL intensity profile with MD, HD, and SEQ data were designated BVSINT_MD_, BVSINT_HD_, and BVSINT_SEQ_, respectively.

## Results

### Analysis using two models based on various marker densities

**Fig. 1:**
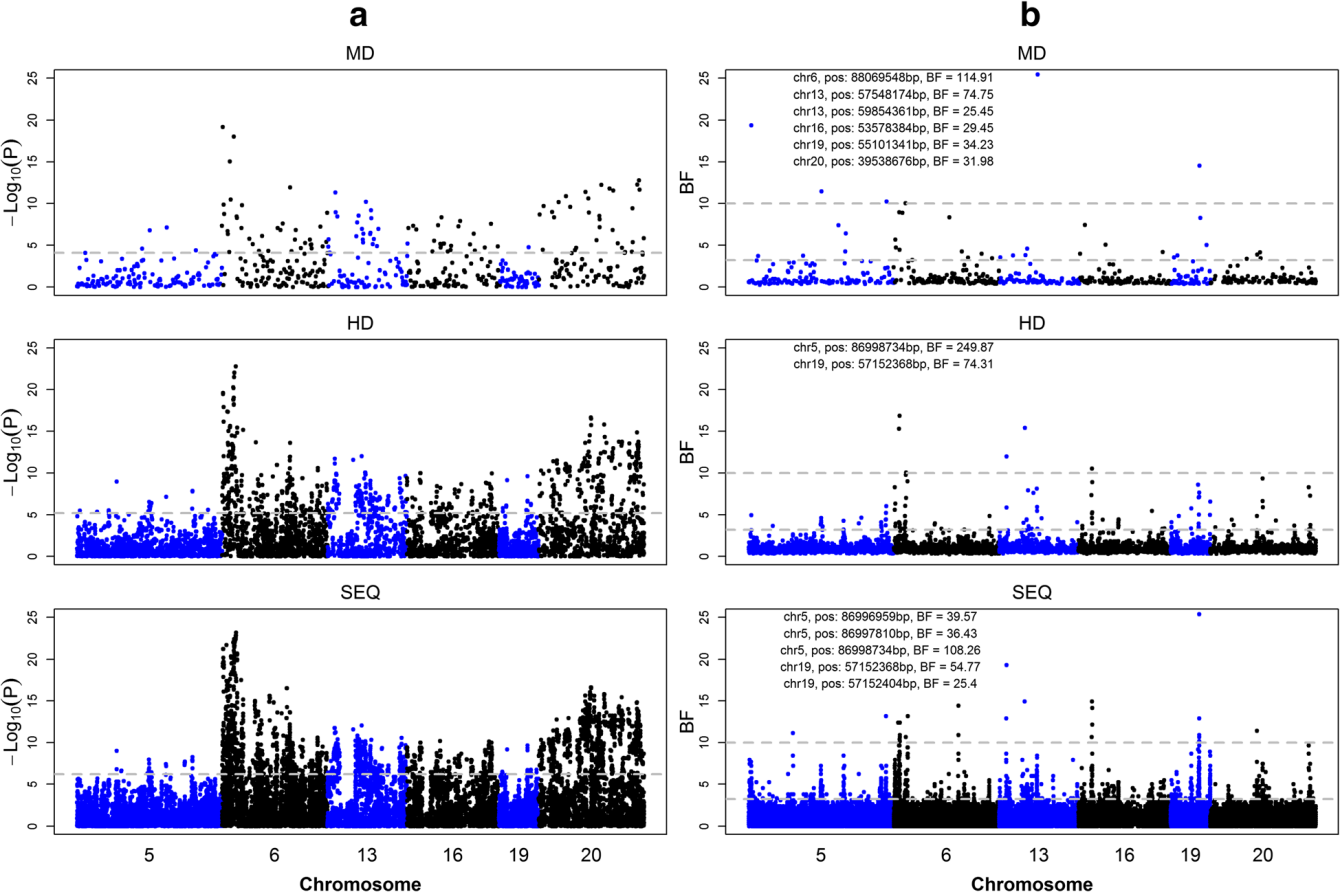
Genome-wide plot for association of SNPs with udder health. (**a**) Plots generated by the LM model based on MD, HD, and SEQ data. (**b**) Plots generated by BVS model based on MD, HD, and SEQ data. Six chromosome regions between 84 and 95 Mb on BTA5, 88 and 96 Mb on BTA6, 57 and 63 Mb on BTA13, 48 and 55 Mb on BTA16, 55 and 58 Mb on BTA19, and 32 and 40 Mb on BTA20 are marked in alternating colors for clarity. The vertical axis is − log_10_(*P*) for the LM and BF for the BVS model, respectively; the horizontal dotted line indicates the genome-wide significance levels (for BVS, 3.2 is considered as putative and 10 is considered as strong evidence); the text on the subfigures presents the significant SNPs that were detected by the BVS model with a BF greater than 25

Figure 1 shows the associations of SNPs with mastitis using the LM and BVS models. For each model, the association patterns were similar for the three marker densities, although the number of significant SNPs decreased with decreasing marker densities, and both models showed peaks at similar locations. However, the BVS model presented clearer signals for QTL regions. In addition, with increasing marker densities, the peaks of putative QTL became sharper for the BVS model and the boundaries of adjacent QTL regions became more obvious for the LM model.

Details on the SNPs detected by LM_MD_, LM_HD_, LM_SEQ_, BVS_MD_, BVS_HD_, and BVS_SEQ_ are in Table S1 [See Additional file 1: Table S1]. Table 1 presents the numbers of significant SNPs that were detected with the LM and BVS models. A total of 26 (MD), 75 (HD), and 465 (SEQ) SNPs were significant for both models. The total genetic variance estimated from the BVS model was equal to 110.20 for the MD data, and the variance explained by the SNPs of the six regions was equal to 13.10 (0.32 from SNPs with small effects and 12.77 from SNPs with large effects). Based on the HD data, the total genetic variance was equal to 113.6, and the variance explained by the SNPs of the six regions was equal to 20.54 (0.35 from SNPs with small effects and 20.20 from SNPs with large effects). Based on the SEQ data, the total genetic variance was equal to 123.70, and the variance explained by the SNPs of the six regions was equal to 30.50 (0.32 from SNPs with small effects and 30.18 from SNP with large effects). Using the LM_HD_, there were 5, 8, 3, 19, 17, and 27 significant SNPs in the six targeted regions with a higher test statistic (− log_10_(*P*)) than the highest test statistic obtained with the LM_MD_. Similarly, using LM_SEQ_, there were 0, 6, 0, 7, 2, and 0 SNPs with a − log_10_(*P*) greater than the highest − log_10_(*P*) achieved with the LM_HD_ analysis for the six targeted regions.

**Table 1:** Number and percentage of significant SNPs

Marker	Model	
	LM	BVS
MD	120 (20.4 %)	43 (7.3 %)
HD	967 (12.4 %)	131 (1.7 %)
SEQ	7209 (9.1 %)	1052 (1.3 %)

The analysis was implemented by the linear mixed model (LM) and the Bayesian variable selection model (BVS); figures in brackets are percentages of significant SNPs out of the total number of SNPs used in the analysis

**Fig. 2:**
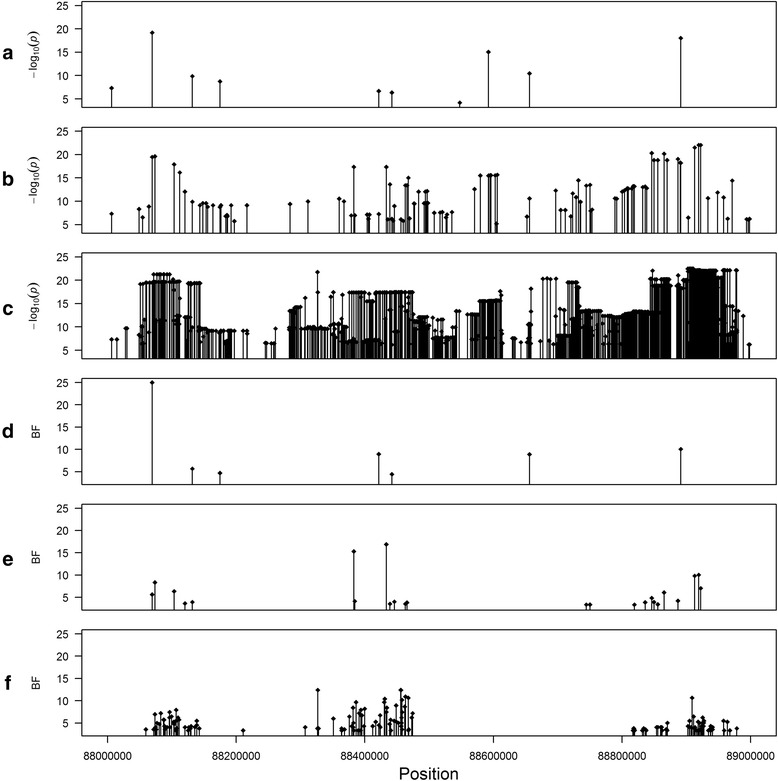
Positions of the significant SNPs on chromosome 6 between 88 and 89 Mb. **a**, **b** and **c** refer to the plots obtained with the LM model based on MD, HD, and SEQ data, respectively, while **d**, **e** and **f** refer to the plots obtained with the BVS model based on MD data, HD, and SEQ data, respectively. The horizontal axis indicates the position of the SNPs. The filled diamond indicates the genome-wide significant SNPs obtained with the LM and BVS (BF > 3.2) models. The SNP with a BF or − log_10_(*P*) greater than 25 was set at 25

The BVS model detected fewer significant SNPs in the six targeted regions than the LM model. Figure 2 graphically represents the position of significant SNPs on BTA6 using both models and the three marker densities. The LM model included one SNP for each run. Therefore, the − log_10_(*P*) values were consistent for the three marker densities with those of the LM model. However, when using the BVS model, the results among the three SNP datasets were not consistent. We observed three cases: (1) seven SNPs were detected by both BVS_MD_ and BVS_HD_, while 70 SNPs were detected by both BVS_HD_ and BVS_SEQ_; (2) some SNPs were only detected by BVS_SEQ_, e.g. the SNP at position 88 326 909 bp on BTA6, which is due to the fact that these SNPs were not present in MD and HD data; and (3) some SNPs were only detected by BVS_MD_, e.g the SNP at position 88 656 290 bp on BTA6, which may be explained by the QTL effect being distributed over several nearby SNPs, probably because the QTL was in strong LD with many markers in the HD and SEQ data.

### Analysis of QTL intensity

**Fig. 3:**
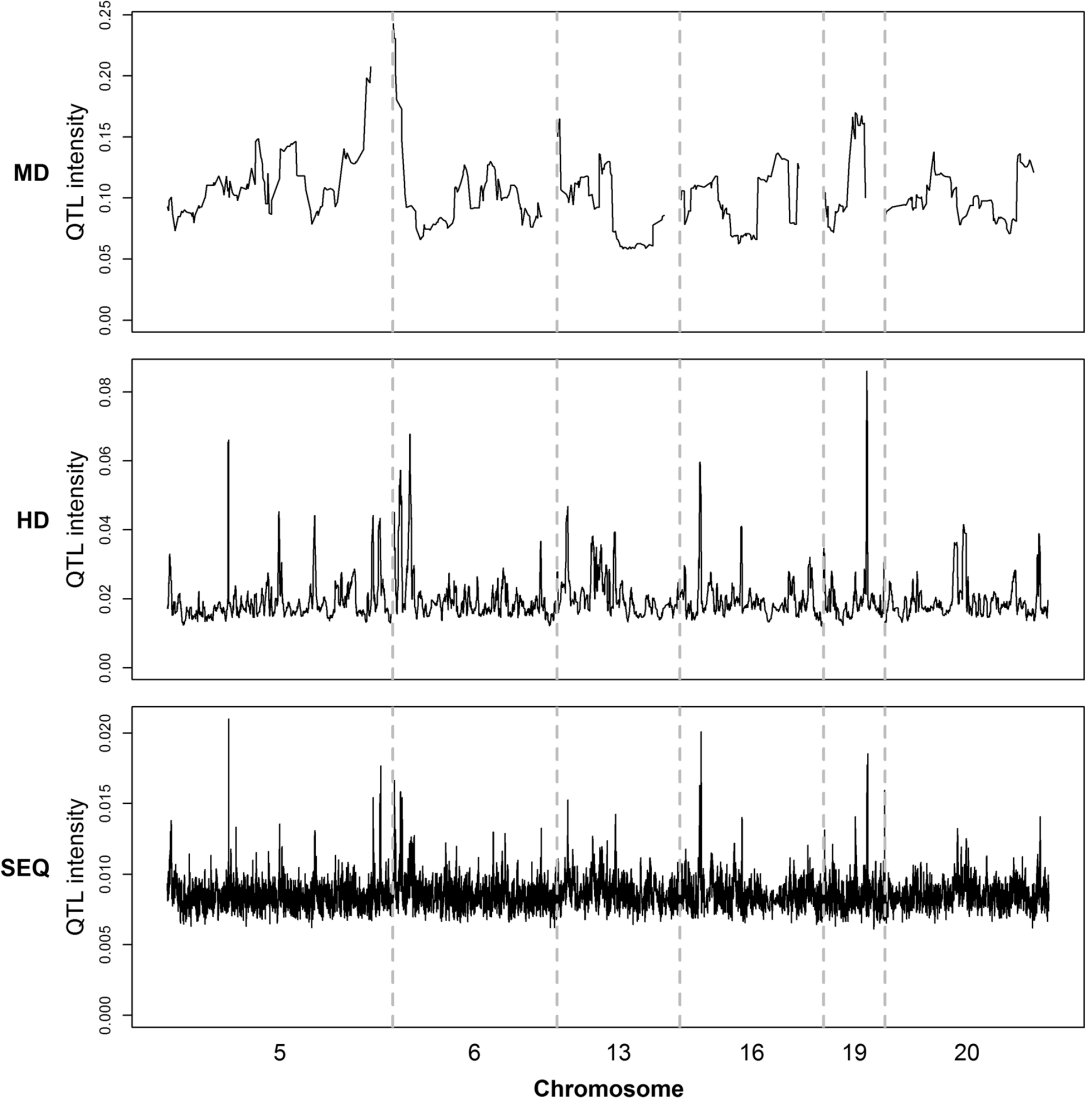
QTL intensity profiles for different marker densities. From top to bottom are shown the plots based on MD, HD, and SEQ data. The six chromosome regions are between 84 and 95 Mb on BTA5, 88 and 96 Mb on BTA6, 57 and 63 Mb on BTA13, 48 and 55 Mb on BTA16, 55 and 58 Mb on BTA19, and 32 and 40 Mb on BTA20; the regions are separated by vertical dotted lines

**Fig. 4:**
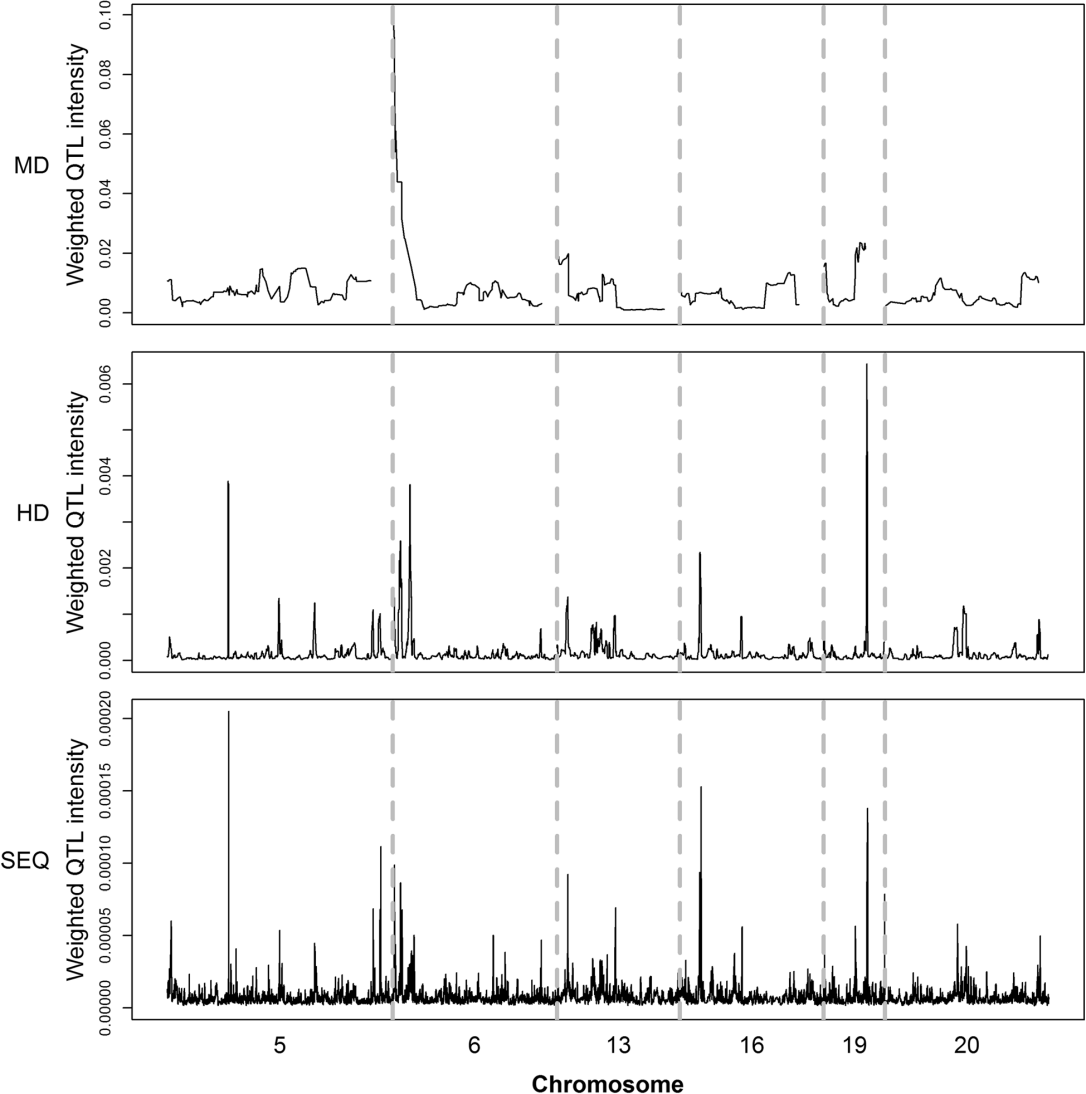
Weighted QTL intensity profiles for different marker densities. From top to bottom are shown the plots based on MD, HD, and SEQ data. The six chromosome regions are between 84 and 95 Mb on BTA5, 88 and 96 Mb on BTA6, 57 and 63 Mb on BTA13, 48 and 55 Mb on BTA16, 55 and 58 Mb on BTA19, and 32 and 40 Mb on BTA20; the regions are separated by vertical dotted lines

**Fig. 5:**
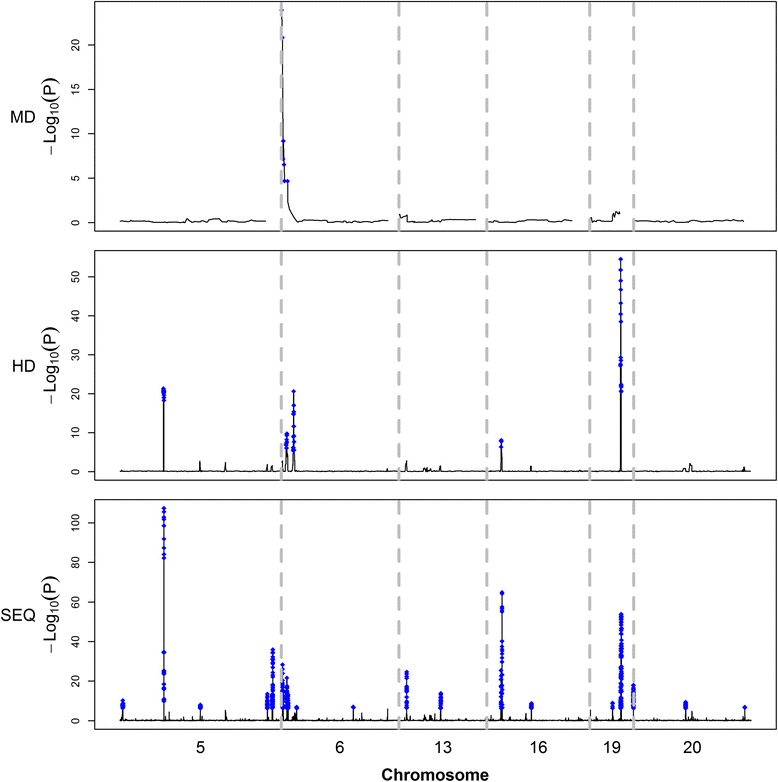
Weighted QTL intensity peaks detected by multiple t-tests for different marker densities. Blue solid circles are the weighted QTL intensities that are significant with the t test. From top to bottom are shown the plots based on MD, HD, and SEQ data, respectively. The six chromosome regions are between 84 and 95 Mb on BTA5, 88 and 96 Mb on BTA6, 57 and 63 Mb on BTA13, 48 and 55 Mb on BTA16, 55 and 58 Mb on BTA19, and 32 and 40 Mb on BTA20; the regions are separated by vertical dotted lines

QTL intensity profiles from the analysis based on the three marker datasets are in Fig. 3. The corresponding weighted QTL intensities are in Fig. 4. The association patterns of the weighted QTL intensities were consistent across the three marker densities. BVSINT_MD_, BVSINT_HD_, and BVSINT_SEQ_ detected one, 16, and 33 QTL intensity peaks, respectively (Fig. 5). Among the SNPs that were significant with the BVS models (BF > 10), 36.4, 87.5, and 86.7 % were within the QTL regions identified by BVSINT_MD_, BVSINT_HD_, and BVSINT_SEQ_, respectively. The positions and intervals of the detected QTL intensity peaks by BVSINT_MD_ and BVSINT_HD_ are in Table 2. BVSINT_MD_ detected only one QTL intensity peak on BTA6. Table 3 shows the 51 genes that were located within or adjacent to the QTL intensity peaks detected by BVSINT_SEQ_. Among these QTL intensity peaks, 27 were located within or overlapped with known genes, while the others were 5 to 165 kb away from the nearest known gene. The average LD (r^2^) of the 33 QTL intensity regions was equal to 0.68. The average interval length of the QTL intensity peaks became smaller as marker densities increased and was approximately 1.39, 0.22, and 0.10 Mb for BVSINT_MD_, BVSINT_HD_, and BVSINT_SEQ_, respectively.

**Table 2:** QTL regions detected by QTL intensity analysis using the MD and HD data

Marker	Chr	Start_pos (bp)	End_pos (bp)	Reg_size (bp)
MD	6	88006286	89399736	1,393,450
HD	5	86941977	87064339	122,362
	5	89391490	89553633	162,143
	5	91099849	91359899	260,050
	5	93966483	94118706	152,223
	5	94289241	94526253	237,012
	6	88006286	88275373	269,087
	6	88283864	88476481	192,617
	6	88808116	88964217	156,101
	13	57446360	57841028	394,668
	13	59741546	60044106	302,560
	16	48939356	49090916	151,560
	16	50915500	51130913	215,413
	19	57101615	57198154	96,539
	20	35703892	36030444	326,552
	20	39473971	39663597	189,626

Chr = chromosome; Start_pos = start position of the QTL region; End_pos = end position of the QTL region; Reg_size = size of the QTL region

**Table 3:** Genes located within or closest to the QTL regions

Chr	Start_pos(bp)	End_pos(bp)	Reg_size	Gene symbol	Distance(bp)	r^2^
5	84128853	84232545	103,692	*SSPN*	overlap	0.51
5	86979804	87132636	152,832	*SOX5,LOC101906017*	overlap	0.36
5	87331630	87387548	55,918	*TRNAG-CCC*	121,327	0.65
5	88917412	88967709	50,297	*LDHB*	overlap	0.83
5	89457697	89510420	52,723	*SLCO1C1*	Overlap	1.00
5	89581859	89643424	61,565	*PDE3A*	Overlap	0.88
5	91175414	91271746	96,332	*PLEKHA5*	Overlap	0.56
5	94025000	94448464	423,464	*DERA*	Overlap	0.31
6	88056115	88164130	108,015	*DCK*	Overlap	0.58
6	88355595	88476214	120,619	*SLC4A4*	Overlap	0.70
6	88813863	88828238	14,375	*GC*	74,566	1.00
6	88841591	89072556	230,965	*NPFFR2*	102,294	0.79
6	92877489	92929724	52,235	*SCARB2, FAM47E*	Overlap	0.62
6	93440325	93546958	106,633	*SOWAHB,SEPT11*	Overlap	0.63
6	95216638	95255224	38,586	*LOC101904933*	Overlap	0.63
13	57509023	57581780	72,757	*EDN3*	Overlap	0.75
13	57733575	57868987	135,412	*SLMO2, LOC100140850*	Overlap	0.99
13	58701953	58817166	115,213	*C13H20orf85*	within	0.78
13	59072545	59132622	60,077	*ZBP1*	within	0.25
13	59150552	59236725	86,173	*CTCFL,LOC101903272*	within	0.41
13	59421380	59480987	59,607	*BMP7*	overlap	0.53
13	59847351	59865177	17,826	*LOC101903611*	18,568	0.97
16	48274699	48326469	51,770	*NPHP4*	overlap	0.64
16	48926813	49072532	145,719	*LOC100297820*	138,439	0.61
16	50624458	50723980	99,522	*LOC515105,WRAP73,TPRG1L, MEGF6*	overlap	0.52
16	51020468	51057724	37,256	*PRDM16*	overlap	0.95
19	55035976	55078482	42,506	*SEPT9*	overlap	0.99
19	56545652	56643735	98,083	*SAP30BP, RECQL5, SMIM5, LOC100302389,MYO15B, LOC101907888*	overlap	0.97
19	57102415	57184032	81,617	*FDXR, USH1G, OTOP2, FADS6*	overlap	0.36
19	57979089	57999724	20,635	*RPL38*	164,726	0.58
20	35525215	35566004	40,789	*OSMR,RICTOR*	overlap	0.66
20	35900432	36010180	109,748	*LIFR,EGFLAM*	overlap	0.67
20	39432294	39613378	181,084	*RAI14*	overlap	0.77

QTL regions were detected by analysis of QTL intensity using SEQ data; Chr = chromosome; Start_pos = start position of the QTL region; End_pos = end position of the QTL region; Reg_size = size of the QTL region; Distance = distance between gene and QTL region; r^2^ = linkage disequilibrium between significant SNPs (BF ≥ 3.2) within each region

## Discussion

To our knowledge, only two studies have performed sequence-based association studies for clinical mastitis [52, 38]. In this study, based on SEQ data and a BVS model for QTL intensity analysis, 33 QTL regions with an average length of 0.069 Mb were detected in the six pre-selected chromosome regions.

### Comparison of the two models

The association patterns between SNPs and mastitis were similar for the LM and BVS models. However, signals were much more distinct when using the BVS model (Fig. 1). This indicates that the BVS model is able to identify and narrow down regions of putative QTL and to distinguish linked QTL.

According to the significance level used to detect SNPs in this study, the number of significant SNPs identified with the BVS model was smaller than with the LM model (see Table 1). The difference in the percentage of significant SNPs between the two models increased with increasing marker densities. This is because the LM model is a single-SNP model and, thus, a QTL effect is determined by a single SNP in each run. Consequently, many SNPs in LD with the QTL can have a significant effect on the model’s output. In contrast, the BVS model estimates the effects of all SNPs simultaneously, and the effect of a QTL may be represented by a single SNP or distributed over several SNPs that are in strong LD with the QTL [53] (i.e., several SNPs could together represent the effect of a single QTL).

### Comparison of the three marker densities

In a previous study, using a simulated dataset, Cleveland et al. [53] reported that Bayesian analysis showed clear QTL signals when SNPs were clustered in a 0.5 to 5.0 cM region. In this study, three marker densities were analyzed, with average distances between markers of 70 kb (for MD), 5.5 kb (for HD), and 0.5 kb (for SEQ).

Our results (Fig. 1) showed that peak locations were largely consistent for the three marker densities. Spencer et al. [54] reported that marked differences in genome coverage may not translate into appreciable differences in power to detect causative variants when using an additive model. However, it is obvious that there were fewer markers near a QTL in the MD data compared to the HD data, and therefore, there were few markers in LD with a QTL [53].

Since, in this study, a single-SNP regression was performed using the LM model and all lower-density SNPs were included in the higher-density dataset, all significant SNPs that were identified with LM_MD_ were also detected with LM_HD_, and all significant SNPs that were identified with LM_HD_ were also detected with LM_SEQ_ if the same significance threshold was used. However, some SNPs that were detected with LM_MD_ did not reach significance with LM_HD_, and some SNPs detected with LM_HD_ did not reach significance with LM_SEQ_, because of the different significance thresholds for the three marker densities as a result of Bonferroni multiple-testing correction. In addition, as density increased, the LD within a given region became stronger and a larger number of markers around a QTL could show an effect on the trait. Therefore, increasing marker density does not necessarily clarify the QTL region boundaries when using LM analysis. In addition, there were 79 significant SNPs for LM_HD_ with a − log_10_(*P*) that was larger than the highest − log_10_(*P*) obtained with LM_MD_ for the six regions, but only 15 significant SNPs for LM_SEQ_ with a − log_10_(*P*) that was larger than the highest − log_10_(*P*) obtained with LM_HD_ for the six regions. Thus, the relative increase in associated SNPs from the HD to the SEQ data was less than the increase from the MD to the HD data. This may be due to imperfect imputation of sequence data due to the small reference size or may reflect that the density of the HD data was sufficient for QTL detection and further density increases only give small improvements. However, it should also be noted that both the MD and HD data have ascertainment biases towards common variants, which are more suitable for GWAS.

In contrast, some significant SNPs in the BVS_MD_ set were not detected when using BVS_HD_ and BVS_SEQ_ data, and some significant SNPs in the BVS_HD_ set were not detected in the BVS_SEQ_ set, although the same BF criterion was used for the three maker densities. Moreover, increasing marker density did not result in a higher BF. Since the BVS model fits all markers simultaneously, it is possible that when markers are very close to a QTL but are not part of low-density data, the QTL effect may be shifted to nearby markers [53], or a QTL effect may be represented by a single SNP when using low-density data but shared by many SNPs when using high-density data. This suggests that instead of a single SNP, a summary statistic across a small region is necessary to detect a QTL when using the BVS model.

In spite of the interesting properties of high-throughput sequencing, it is necessary to take some of the limitations of imputed sequence data into consideration. Only 89 sequenced animals from three breeds were used as imputation reference population and, therefore, imputed sequences were expected to have relatively low imputation accuracy, especially for SNPs with low minor allelic frequencies. Brøndum et al. [42] reported an imputation accuracy of around 0.90 when the reference population included 242 individuals, which is much lower than the accuracy obtained (0.97) in imputation of HD markers from 50 k data for Nordic Holstein cattle [55]. In addition, the errors in calling variants may be higher with high-throughput sequencing than with SNP-array genotyping data [42]. Daetwyler et al. [22] also pointed out that results of association studies that are based on imputed sequences should be interpreted with caution, since SNPs with slightly higher P values than the most significant SNPs from multiple-testing can also be considered as potential causative mutations, particularly if there is strong supporting functional evidence. In addition, with the very high density of SNPs in sequence data, it is expected that a large number of SNPs will be in high or near perfect LD with the causal variants. This makes it difficult to distinguish causal variants from SNPs in high or near perfect LD with the causal variants using a Bayesian approach since the QTL effect may be diluted across many neighboring SNPs. Therefore, in this study, we removed one of the SNPs from a pair in which two SNPs were in complete LD although this may cause the loss of causative variants. However, it is most likely that adjacent SNPs that are in complete LD are close to each other. In other words, if a causative variant is removed by LD-based pruning, it will be replaced by another SNP that is closely associated with the causal variant. Thus in these conditions, the putative loss of causative variants should have little influence on the identification of QTL peaks. In fact, if a neutral SNP is in complete LD with a causal SNP, the latter cannot be differentiated using either the single-SNP linear mixed model or the Bayesian model. However, the Bayesian approach is more appropriate for narrowing QTL regions and distinguishing multiple QTL regions that segregate closely. Post-analysis based on bins using the Bayesian approach without pruning the SNPs may avoid the possible loss of causal variants and dilution of QTL effects.

### QTL regions

In the BVS model, the QTL effect may be distributed over several markers that are in LD with the QTL. Therefore, combining the posterior probabilities of closely located markers can result in higher power when inferring the presence of a QTL [23]. Detection of QTL regions based on QTL density profiles was initially proposed by Sillanpaa and Arjas [29]. Using simulated data, QTL regions are easy to determine because of the simplicity of the simulation [56]. However, when using real data, the significance threshold for QTL intensity peaks is not as well defined because of the low signal to noise ratio (Fig. 4). Previous studies have detected QTL peaks by using QTL intensity, but only a few defined the interval and specific position of QTL regions [34, 56, 48]. In this study, we used multiple t-tests based on weighted QTL intensities to determine QTL regions.

Based on the present data, the association patterns of QTL intensities (Fig. 4) and BF (Fig. 1) were similar. However, the QTL intensities presented clear peaks. Analysis of the QTL intensity profiles detected only one QTL peak on BTA6. This indicates that studies with higher marker densities have greater power to detect QTL regions by QTL intensity profile analysis. In addition, the average length of the detected QTL regions became shorter as marker densities increased (Tables 2 and 3), which suggests that QTL regions can be refined by using high-density markers and applying the BVS model together with a QTL intensity profile.

Based on SEQ data, the average r^2^ (LD) of significant SNPs between a pair of QTL regions was lower than 0.55, while the average r^2^ of significant SNPs within each QTL region (average 0.68) was higher than the r^2^ among different QTL regions. In addition, comparisons between QTL intensity profiles and BF (>10) showed that 87 % of the significant SNPs detected by BF were located within the QTL regions determined by QTL intensity profiles based on the HD and SEQ data. Among the 33 QTL regions detected by QTL intensity profiles using the SEQ data, 27 regions contained a known gene. For the remaining six regions, the closest gene was at most 165 kb away.

Five of the detected regions have been reported by previous studies or contain genes that are known to be functionally associated with mastitis traits. One of these QTL regions (between 88 056 115 and 88 164 130 bp on BTA6) overlaps with the *deoxycytidine kinase* (*DCK*) gene. Abdel-Shafy et al. [57] reported that this gene is associated with somatic cell score, which is a mastitis indicator [58]. Another QTL region (between 88 355 595 and 88 476 214 bp on BTA6) overlapped with the s*olute carrier family 4, sodium bicarbonate co-transporter, and member 4* (*SLC4A4*) gene belonging to the *SLC4* family. Sodeland et al. [59] reported a high correlation of SNPs surrounding the *SLC4A4* gene with clinical mastitis. A third QTL region between 88 984 167 and 89 072 556 bp on BTA6, overlapped with a previously reported QTL region between 89 and 91 Mb for mastitis [60]. This region contains a part of the *neuropeptide FF receptor 2* (*NPFFR2*) gene that encodes a member of a G-protein-coupled neuropeptide receptors subfamily that is activated by the neuropeptides A-18-amide (NPAF) and F-8-amide (NPFF) [61]. Sun et al. [61] showed that NPFF is involved in anti-inflammatory effects, both *in vitro* and *in vivo,* and Minault et al. [62] showed that it modulates the proliferation of human T lymphocytes. In addition, Sodeland et al. [59] detected a QTL region around 35.5 Mb on BTA20 which is associated with clinical mastitis. A fourth QTL region, between 35 900 432 and 36 010 180 bp on BTA20, was detected and overlapped with the *leukemia inhibitory factor receptor alpha* (*LIFR*) gene. LIFR is a breast cancer metastasis suppressor upstream of the Hippo-YAP pathway and a prognostic marker [63]. Finally, a QTL region between 57 509 023 and 57 581 780 bp on BTA13 overlapped with the QTL at 57.54 Mb reported by Sahana et al. [64]. This region overlaps with the *endothelin* 3 (*EDN3*) gene, which influences neutrophil activation [65]. In blood, neutrophils are the major leukocytes that respond to inflammatory stimuli. Therefore, *NPFFR2*, *SLC4A4*, *DCK*, *LIFR*, and *EDN3* are candidate genes for susceptibility to mastitis.

## Conclusions

The power of QTL detection can be increased by increasing marker densities and the BVS model outperforms the LM model in refining QTL locations with clear boundaries between linked QTL. Based on the results obtained with the SEQ data, six preselected regions were refined into 33 candidate QTL regions for udder health. Furthermore, the comparison between these candidate QTL regions and known genes suggests that *NPFFR2*, *SLC4A4*, *DCK*, *LIFR*, and *EDN3* may be considered as candidate genes for mastitis susceptibility. Further studies are required to validate the causative loci that underlie these QTL and to investigate the function of the candidate genes that affect udder health.

## Additional file

Additional file 1: Table S1. Position of detected SNPs by LM_MD_, LM_HD_, LM_SEQ_, BVS_MD_, BVS_HD_, and BVS_SEQ_. **Table S1.** contains six sheets that provide the position of significant SNPs detected by LM_MD_, LM_HD_, LM_SEQ_, BVS_MD_, BVS_HD_, and BVS_SEQ_.
